# Supplementary material for: Lonicera caerulea Extract Attenuates Non-Alcoholic Fatty Liver Disease in Free Fatty Acid-Induced HepG2 Hepatocytes and in High Fat Diet-Fed Mice
Source: Nutrients. 2019 Feb 26;11(3):494. doi: 10.3390/nu11030494 (PMC6471428; doi:10.3390/nu11030494)
Supplement: Supplementary file 1 [file nutrients-11-00494-s001.pdf]

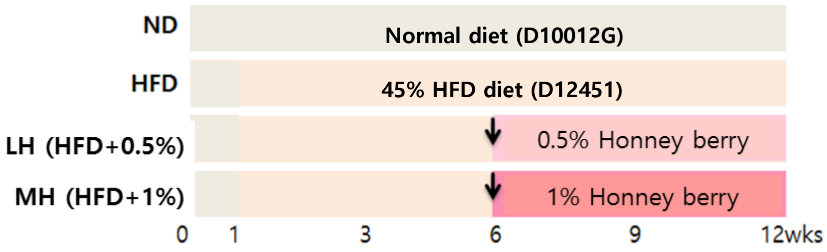

Supplement figure 1. Schematic overview of the experimental design.

Supplement Table 1. Calculated diet composition of HFD groups.

Class description	Ingredient	HFD	HFD with 0.5% HBE	HFD with 1% HBE
Protein	Casein, Lactic, 30 Mesh	23.3	23.2	23.1
Protein	Cystine, L	0.3	0.3	0.3
Carbohydrate	Sucrose, Fine Granulated	20.6	20.5	20.4
Carbohydrate	Lodex 10	11.7	11.6	11.5
Carbohydrate	Starch, Corn	8.5	8.4	8.4
Fiber	Solka Floc, FCC200	5.8	5.8	5.8
Fat	Lard	20.7	20.6	20.5
Fat	Soybean Oil, USP	2.9	2.9	2.9
Mineral	S10026B	5.8	5.8	5.8
Vitamin	Choline Bitartrate	0.2	0.2	0.2
Vitamin	V10001C	0.1	0.1	0.1
Dye	Dye, Red FD&C #40, Alum. Lake 35-42%	0.0	0.0	0.0
	**Honeyberry Extract (HBE)**	**0**	**0.5**	**1**
	**Total (g)**	**100**	**100**	**100**
